# Utility and impact of magnetic resonance elastography in the clinical course and management of chronic liver disease

**DOI:** 10.1038/s41598-024-51295-1

**Published:** 2024-01-20

**Authors:** Colin Feuille, Swathi Kari, Roshan Patel, Rohan Oberoi, Jonathan Liu, Michael Ohliger, Mandana Khalili, Michele Tana

**Affiliations:** 1 Division of Gastroenterology and Hepatology, Department of Medicine, University of California San Francisco, San Francisco, CA USA; 2 Department of Hospital Medicine, Gould Medical Group, 600 Coffee Road, Modesto, CA 95355 USA; 3 Department of Radiology and Biomedical Imaging, University of California San Francisco, San Francisco, CA USA; 4 The Liver Center, University of California San Francisco, San Francisco, CA USA; 5 Data Science Division, Chan-Zuckerberg Biohub, San Francisco, CA 94158 USA; 6 Present Address: Department of Gastroenterology, The Permanente Medical Group, Walnut Creek, CA USA; 7 Present Address: Center for Family Medicine, University of North Dakota School of Medicine and Health Sciences, Minot, ND USA

**Keywords:** Hepatology, Liver fibrosis, Medical research

## Abstract

We aimed to characterize scenarios where magnetic resonance elastography (MRE) of the liver was ordered and its impact on clinical course and management. 96 consecutive MRE examinations and subsequent encounters over 14 months were reviewed. Indication for MRE of the liver and subsequent management were abstracted from the medical record. In all cases, non-invasive assessment of liver fibrosis was the primary indication and at least one additional rationale was noted. There was a significant decrease in recommendations to undergo liver biopsy after MRE. Additionally, a greater percentage of those recommended to undergo biopsy completed the procedure after discussion of the results. Given the significant cost and rare but serious risks of liver biopsy, MRE of the liver provides an attractive, safer alternative that may have a comparable impact on management, or select cases where biopsy is essential to guide management. We demonstrate the versatility of MRE in real-world hepatology practice, including its utility as a non-invasive surrogate for liver biopsy.

## Introduction

While considered gold standard to determine stage of liver fibrosis, biopsy is limited by sampling error, subjective interpretation, and complications including bleeding, and rarely, death ^1–4^. Therefore, noninvasive technology such as magnetic resonance elastography (MRE) has in some cases replaced biopsy to evaluate disease severity. MRE detects propagation of acoustic shear waves through the liver to generate quantitative cross-sectional maps of liver stiffness, and has excellent sensitivity and specificity, with a c-statistic of at least 0.9 for detecting advanced fibrosis ^5^. While advantages of MRE are well known, its role in current practice is less established, and there is scarce data on how results impact clinical management. This technology may be particularly helpful for underserved populations with increased risk of health disparity and understanding its impact may aid in developing strategies to address care gaps. We aimed to characterize clinical scenarios where MRE was ordered at our urban county hospital and assess the results’ impact on subsequent management.

## Methods

96 consecutive MRE examinations between July 1, 2016 and August 31, 2017 were reviewed. At our institution, when the clinical concern is diffuse liver disease, all MR examinations included T2-weighted imaging with and without fat saturation, T1-weighted chemical shift imaging, diffusion weighted imaging, proton density fat fraction (PDFF) quantification, iron quantification (R2*), and elastography. For some indications, such as screening for hepatocellular carcinoma (HCC), T1-weighted imaging before and after gadolinium administration was also performed. All exams were performed on a 3.0 T wide-bore MRI scanner (Somatom Skyra, Siemens Medical) according to standard protocol. A pneumatic driver (Resoundant, Inc) was attached to the abdomen and operated at 60 Hz. Axial plane gradient-echo images in four slices were obtained using motion sensitizing gradients at four locations within the liver. Stiffness maps were generated in standard fashion together with maps indicating areas of high (> 95%) confidence. In routine clinical interpretation, a radiologist placed regions-of-interest to measure liver stiffness, documenting both mean and range stiffness in kilopascals (kPa). In addition, fibrosis was graded per institutional practice: (1) no fibrosis (mean stiffness < 3 kPa); (2) mild-moderate fibrosis, grade 1–2 (mean stiffness 3–4.5 kPa); or (3) advanced fibrosis, grade 3–4 (mean stiffness > 4.5 kPa).

Demographic information recorded included age, gender, race, BMI, and insurance status. Laboratory data closest to the MR date was used, to a maximum of 6 months prior. If no pre-MR laboratory data was available, the closest data within 6 months after MR was recorded, except if treatment was changed based on the MR results. We reviewed all documented indications for each MR and grouped these into 12 categories. Encounters after MR were reviewed for evidence of how results influenced management, and management plans were grouped into 13 categories. These categories were generated after holistic review of all medical records in the study period, and agreement between two authors (CF and MT) on common language that could be applied to scenarios described in the patient encounters.

To determine whether pre- and post-MR patients differed on need for liver biopsy, we performed a two-tailed z-test. Specifically, we assumed that the liver biopsy recommendation status of an individual was a Bernoulli random variable with probability P. Our null hypothesis was that P_0_ was equal to 0.47, the measured fraction of patients who were recommended liver biopsy pre-MR.

Because the sample size was large (N = 96), the null hypothesis’ distribution of the number of liver biopsy recommendations NP_0_ could be approximated with a normal distribution with variance NP_0_(1 − P_0_). We could thus perform a two-tailed z test on the post-MR biopsy probability P_1_. The calculated z-score was then given by (NP_1_ − NP_0_)/sqrt(NP_0_(1 − P_0_)).

All methods were carried out in accordance with relevant guidelines and regulations. All experimental protocols were approved by the UCSF Institutional Review Board. The same IRB (UCSF) approves the waiver or alteration of informed consent described in the application.

## Results

**Figure 1:**
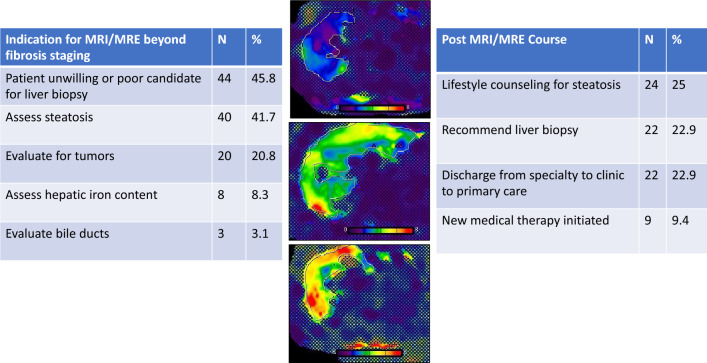
Clinical scenarios in which MRE was ordered and impact on subsequent clinical course. The primary indication for MRE in all cases was assessment of liver fibrosis, and at least one additional indication was identified in every case. Management plans after MRE are also presented; in many cases, multiple management courses were applicable. Stiffness maps (elastograms) were created by Michael Ohliger using Siemens MR Elastography software, syngo MR D11D (https://www.siemens-healthineers.com/en-us/magnetic-resonance-imaging/options-and-upgrades/clinical-applications/mr-elastography).

Patients were median age 57 years (range 44–70), 51% female, and 50% Asian/Pacific Islander (Table 1). MRE was ordered for patients with a wide variety of etiologies of liver disease, including chronic hepatitis B (24, 30.4%), chronic hepatitis C (3, 3.8%), metabolic dysfunction-associated steatotic liver disease (28, 35.4%), autoimmune liver disease (3, 3.8%), iron overload (1, 1.3%). 11 patients (13.9%) had more than one disease (13.9%), and 9 patients (11.4%) had other/unknown liver diseases. Only 3 (3.8%) of the MRE exams were ordered by primary care providers, with the remaining 93 (96.9%) ordered by gastroenterology or hepatology providers. In all cases, non-invasive assessment of fibrosis was a primary indication for ordering MRE, and in every case at least one additional rationale for ordering a liver MR was noted (Fig. 1). The clinician sought to assess steatosis via proton density fat fraction in 40 cases (42% of studies), screen for hepatocellular carcinoma in 11 cases (11.8%), and assess hepatic iron content in 8 cases (8.6%). Evaluating a focal liver lesion, investigating an elevated alpha-fetoprotein, and assess bile ducts were additional indications for obtaining the MRE in 5 (5.4%), 4 (4.3%), and 3 (3.2%) patients, respectively.

**Table 1:** Patient clinical characteristics (n = 96).

Demographic data	
Median age (SD)	57.5 (13.2)
Gender	
Male % (SD)	49
Female % (SD)	51
Race	
Caucasian %	12.5
African %	11.5
Hispanic %	25
Asian/Pacific Islander %	50
Unknown %	1
Body mass index (SD)	26.5 (5.69)
Insurance	
Public %	57.3
Private %	39.6
Uninsured %	3.1
Cormorbidities	
Diabetes mellitus %	42.7
Hypertension %	49
Dyslipidemia %	52.1
LABS	
Plts(SD)	206 (69.8)
HgbA1c % (SD)	5.6 (1.4)
Creatinine mg/dL (SD)	0.79 (0.86)
Albumin g/dL (SD)	4.4 (0.32)
T. Bili mg/dL (SD)	0.6 (0.42)
ALP IU/L (SD)	92 (33.0)
AST IU/L (SD)	34 (38.6)
ALT IU/L (SD)	37 (76.3)
INR	1.0 (0.13)
HBV DNA IU/ML	8897
Labortory-based fibrosis scores	
APRI (SD)	0.358 (0.582)
FIB4 (SD)	1.52 (1.71)

The MRE technical success rate was 98%. The mean liver stiffness measurement (LSM) by MRE was 2.66 kPa (range 1.5–10.0 kPa). 74% of patients had no significant fibrosis, 20% had mild-moderate fibrosis, and 6% had advanced fibrosis based on LSM (Table 2).

**Table 2:** MR elastography and abdominal MRI results.

MR elastography results	
Mean liver stiffness measurement (SD)	2.66 kPa (1.22)
No fibrosis (mean LSM < 3 kPa)	74%
Mild-moderate (grade 1–2) fibrosis (mean LSM 3–4.5 kPa)	20%
Advanced (grade 3–4) fibrosis (mean LSM > 4.5 kPa)	6%
Mean proton density fat fraction (SD)	7.5% (5.4%)

Additional MRI findings	N (%)
Focal liver lesions	6 (6.25)
Splenomegaly	6 (6.25)
Nodularity	4 (4.16)
Varices	3 (3.13)
Ascites	2 (2.08)
Portal hypertension	1 (1.04)
Dilated portal vein	0 (0)

Prior to MRE, 44 patients (45.8% of the study population) had been recommended to undergo liver biopsy but declined the procedure or were considered poor candidates. After MRE, biopsy was recommended for 22 patients (22.9% of cases), a significant decrease (p = 0.03). 15 of the 22 patients (68%) agreed to biopsy after being presented with the MRE results. 7 of those patients ultimately underwent liver biopsy during the follow-up period. Of note, biopsy was recommended prior to the MRE but not after reviewing the MRE results for 21 patients (21.9%). Conversely, biopsy was not initially recommended but subsequently recommended after the MRE for 7 patients (7.3%). For 15 patients (15.6%), the recommendation to undergo biopsy remained unchanged before and after the MRE was performed. In 12 cases (12.5%), elastography indicated no significant fibrosis, yet biopsy was still recommended after the MRE. For example, the biopsy was recommended to assess for histologic evidence of steatohepatitis or to clarify the etiology of abnormal transaminase levels.

The most common management after MRE was determination that continued clinical follow-up was appropriate (n = 38, 39.6%). Emphasis on lifestyle modifications was frequently documented after an exam showing steatosis (n = 24, 25%), particularly when steatosis was not previously suspected, as in patients with known chronic viral hepatitis (Fig. 1). After obtaining the MRE results, 22 patients (22.9%) were discharged from specialty care for monitoring in the primary care setting. 9 (9.4%) patients were prescribed a new therapy for their liver disease, based on the MRE results. Variceal and HCC screening recommendations were modified based on the MRE results for 2 patients (2.1%). Unfortunately, 13 patients (13.5%) did not return to clinic after the MRE was performed and were lost to follow-up.

Using the measured probabilities P_0_ = 0.47 and P_1_ = 0.24 yielded a negligible p-value of 6 × 10^–6^ for the two-tailed z-test, which was statistically significant. This indicated that post-MR biopsy recommendation probabilities were significantly different from pre-MR biopsy recommendation probabilities.

## Discussion

For patients with CLD, assessing liver fibrosis is essential for risk stratification and management, and MRE performs well for this purpose ^5^. In current practice, MRE is often performed in the context of a full liver MRI (with or without contrast), which may answer multiple clinical questions with a single study. Our data demonstrate wide ranging clinical benefits that can be derived from the exam, where the fibrosis stage provided by elastography is supplemented by additional data from the MR sequences, PDFF, R2*, and/or MRCP. Coupled with laboratory data, liver MRI with elastography, PDFF, and R2* may facilitate determination of CLD etiology and stage with reasonable certainty, acting as a kind of virtual liver biopsy. For example, in cases with a known cause of CLD such as viral hepatitis, liver MRI with PDFF is helpful to diagnose superimposed steatosis, with elastography providing additional information on fibrosis stage. Likewise, when biochemical workup for abnormal transaminases has narrowed the differential diagnosis to metabolic dysfunction-associated steatotic liver disease or autoimmune hepatitis, MRI with PDFF can determine if steatosis is a factor while elastography stages the degree of hepatic fibrosis.

This strength was evident in our population, where the number recommended to undergo biopsy approximately halved after the exam, suggesting it often provided enough information that biopsy was no longer deemed necessary. Additionally, a greater percentage of those recommended to undergo biopsy completed the procedure after discussion of the results. Given the significant cost and rare but serious risks of biopsy, MRI provides an attractive, safer alternative that may have a comparable impact on management, or select cases where biopsy is essential to guide management. There is increasing interest in abbreviated MR protocols that consist of MRE alone, which may decrease cost and increase availability of the exam. This effort has been aided by a separate CPT code for MRE alone, which was not in use during the study period ^6,7^.

Our study has several limitations, including the lack of transient elastography (FibroScan) data for comparison. While many hepatology practices make frequent use of transient elastography to estimate liver fibrosis, our center does not have this somewhat costly tool. Paradoxically, our deficiency of transient elastography has led to our use of MRE to estimate liver fibrosis and experience with its clinical utility described here.

In conclusion, this study demonstrates that liver MRI with elastography is a valuable, versatile tool to advance the care of patients with CLD.

## Data Availability

The datasets used and/or analyzed during the current study are available from the corresponding author on reasonable request.
